# A Perioperative eHealth Program to Enhance Postoperative Recovery After Abdominal Surgery: Process Evaluation of a Randomized Controlled Trial

**DOI:** 10.2196/jmir.8338

**Published:** 2018-01-02

**Authors:** Eva van der Meij, Judith AF Huirne, A Dorien ten Cate, Hein BAC Stockmann, Piet C Scholten, Paul HP Davids, H Jaap Bonjer, Johannes R Anema

**Affiliations:** ^1^ Amsterdam Public Health Research Institute Department of Public and Occupational Health VU University Medical Center Amsterdam Netherlands; ^2^ Department of Obstetrics and Gynaecology VU University Medical Center Amsterdam Netherlands; ^3^ Department of Obstetrics and Gynaecology Spaarne Gasthuis Haarlem Netherlands; ^4^ Department of Surgery Spaarne Gasthuis Haarlem Netherlands; ^5^ Department of Obstetrics and Gynaecology Diakonessenhuis Utrecht Netherlands; ^6^ Department of Surgery Diakonessenhuis Utrecht Netherlands; ^7^ Department of Surgery VU University Medical Center Amsterdam Netherlands

**Keywords:** telemedicine, cholecystectomy, surgical procedures, operative, perioperative care, convalescence, process assessment

## Abstract

**Background:**

Electronic health (eHealth) interventions have proven effective, but implementation in clinical practice is difficult. More research focusing on the implementation process of eHealth interventions is necessary.

**Objective:**

The objective of this study was to describe the process evaluation of a perioperative eHealth intervention, aiming to enhance recovery after laparoscopic abdominal surgery.

**Methods:**

A process evaluation was carried out alongside a multicenter randomized controlled trial. Patients aged between 18 and 75 years who were scheduled for a laparoscopic cholecystectomy, hernia inguinal surgery, or laparoscopic adnexal surgery were included. The eHealth intervention comprised a website and mobile phone app with the possibility to develop a personalized convalescence plan, a section with information about the surgical procedure and the recovery period, the possibility to ask questions via an electronic consultation (eConsult), and an activity tracker. The process evaluation was carried out using the model of Linnan and Steckler, measuring components such as reach, dose delivered, dose received, fidelity, and participants’ attitudes. Implementation scores were calculated based on the average of the four components. Quantitative data were collected by means of an electronic questionnaire, a logistic database, a weblog, and medical files. Qualitative data were collected by conducting interviews with a subsample of the study participants.

**Results:**

A total of 344 of the 863 eligible patients were included in the study, which accounted for a reach of 39.9%, and 173 participants were randomized to the intervention group. The implementation scores of the different functions of the intervention ranged between 60% and 65%. The website, mobile phone app, and activity tracker were rated 7.3 to 7.6 on a scale of 1 to 10. Almost all participants who were interviewed about the eConsult function rated it as being of additional value if combined with the usual care but not as a replacement for usual care.

**Conclusions:**

Although participants were overall satisfied with the intervention, the implementation scores of the different functions of the intervention were fair. More research is needed to evaluate the barriers and facilitators for implementation of this perioperative eHealth intervention in normal practice outside study setting.

**Trial Registration:**

Netherlands Trial Registry NTR4699; http://www.trialregister.nl/trialreg/admin/rctview.asp?TC=4699 (Archived by WebCite at http://www.webcitation.org/6vr02V4KK)

## Introduction

The conviction that patients heal better in their own personal environment has been one of the main drivers reducing hospital stay after surgical interventions. Cutting direct hospitalization costs while increasing revenues due to more efficient use of hospital resources is another strong incentive. However, the transition from in-hospital recovery to domestic convalescence has occurred at a high pace without sufficient attention to the needs of patients [1]. As a consequence, the length of recovery after surgery takes longer than the period considered to be needed from a medical perspective [2-6]. Literature shows that patients deal with feelings of uncertainty regarding their recovery when they are at home, and in addition, it is proven that influencing these feelings by education and support would have a positive effect on the length of recovery [6]. Therefore, an electronic health (eHealth) intervention focusing on information supply and guidance during the perioperative period of commonly applied gynecological surgical procedures was developed [7,8]. The effectivity of the eHealth intervention was evaluated in two different trials; patients who used the eHealth intervention in the perioperative period returned to work earlier, reported higher quality of life scores, and lower pain scores than patients who received usual perioperative care only [7,9]. Therefore, the intervention was further developed; new features, such as a mobile phone app, an activity tracker, and an electronic consultation (eConsult) function, were added. In addition, the intervention was extended, whereby it could also be used in the perioperative period of commonly applied general surgical procedures [10]. Due to these promising results regarding the effectivity of the intervention, implementation of the intervention in clinical practice seems logical. However, although literature shows in general that eHealth interventions can show beneficial effects, execution of these types of interventions in clinical practice has often been slower and more difficult than expected [11,12]. To evaluate whether the eHealth intervention was executed as planned, we conducted a process evaluation. The aim is to systematically analyze the process from offering the different aspects of the intervention to the participant. By doing this, the feasibility of the intervention will be investigated and barriers and facilitators for future implementation could be explored. In addition, evaluating the adherence to the intervention protocol should be an integral part of evaluating this type of interventions, as this will play an important role in interpreting the results regarding the effectivity.

## Methods

### Trial Design

This process evaluation was carried out alongside a multicenter randomized clinical trial in seven teaching hospitals in the Netherlands (Netherlands Trial Registry NTR4699). A detailed description of the study design has been published earlier in this journal [10]. The study was reported in accordance with Consolidated Standards of Reporting Trials of Electronic and Mobile HEalth Applications and onLine TeleHealth (CONSORT-EHEALTH) [13] and was approved by the local medical ethics committee under the registration number 2014.301.

### Participants

Patients aged between 18 and 75 years who were scheduled for a laparoscopic cholecystectomy, hernia inguinal surgery, or laparoscopic adnexal surgery were eligible to participate. A sample size calculation was performed; a total of 308 participants would be required. More details about the study population and sample size calculation have been described in the study protocol [10].

### Interventions

Participants were randomized to the control group or the intervention group. Participants from the control group received the usual care and access to a placebo website (containing the patient information brochure about the surgical procedure). Participants from the intervention group received access to the eHealth care program consisting of a website, a mobile phone app, and an activity tracker. The most important tools of the website were the possibility to develop a personalized convalescence plan and the possibility to ask questions to the health care professional (eConsult). Because participants were provided with the option to ask questions via the website by an eConsult, they were initially only offered a telephonic appointment instead of an appointment in the outpatient clinic [10].

### Study Settings

Quantitative data were collected 3 months after surgery by an electronic questionnaire, a logistic database, a weblog, and medical files. In addition, qualitative data were collected by conducting telephone interviews. By means of purposive sampling, a sample of participants was selected from the total study population for an additional interview to collect some additional information for this study. The sample consisted of a subsample of participants who used the eConsult function and a subsample of participants who did not. First, a patient who used the eConsult function was selected, and when this patient was willing to be interviewed, a participant who did not use the eConsult function was selected and matched according to age, gender, and type of surgical procedure. This was repeated until data saturation was reached. This approach enables the exploration of the opinion of the participants about the intervention, including reasons for using or not using the eConsult.

**Figure 1:**
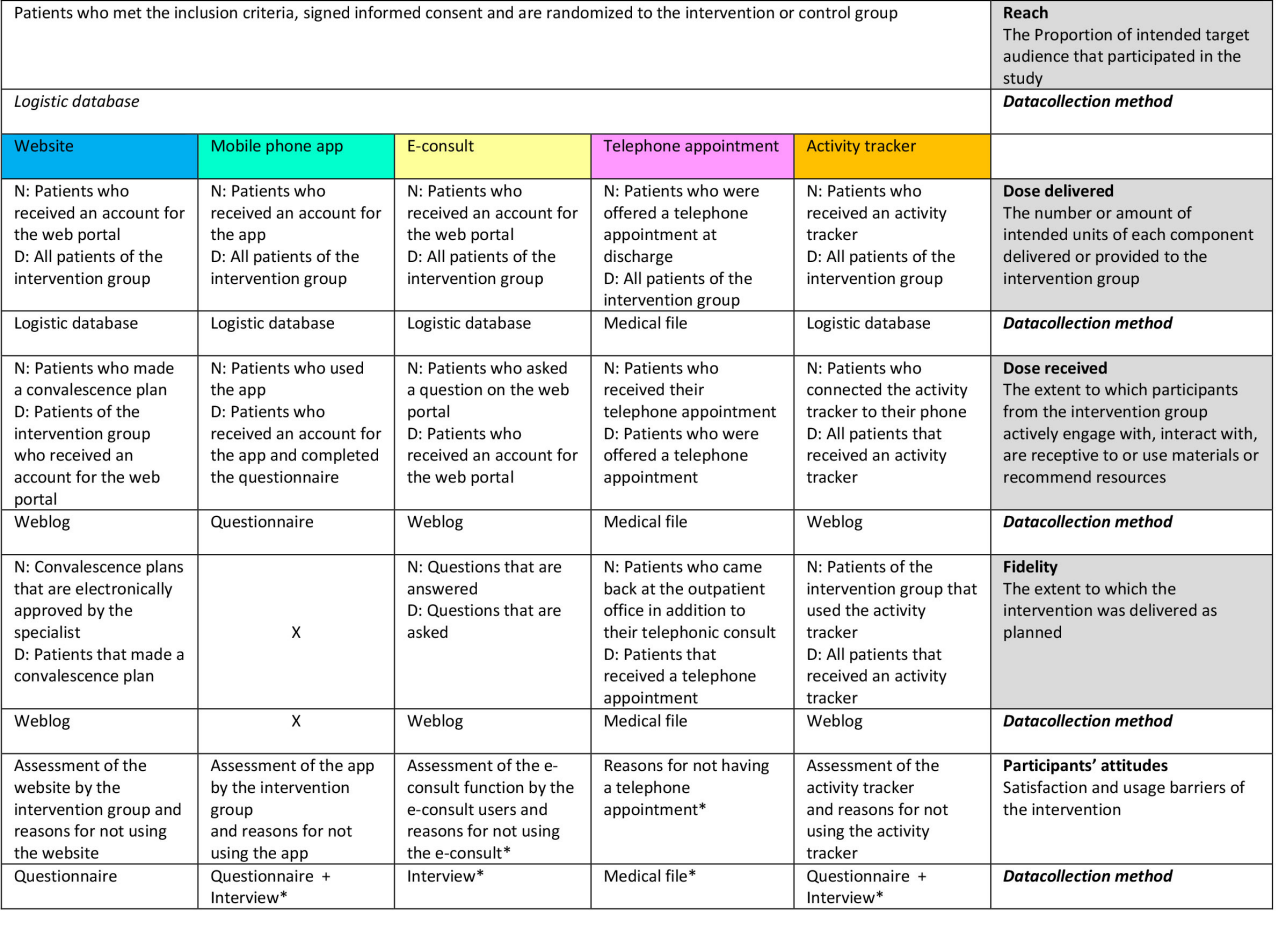
Description of the outcome measures . N: nominator; D: denominator. Asterisk indicates data collection methods not described in the original protocol.

### Outcomes

The process evaluation was performed using the model of Linnan and Steckler [14]. This is a commonly used model in this research field and has the potential to evaluate the process of the implementation systematically because it describes the adherence to the intervention in 5 terms: reach, dose delivered, dose received, fidelity, and participants’ attitudes. Except for the reach component of the model, the components were assessed for each function of the intervention separately. The definitions of the different components of the model are presented in Figure 1. A detailed description of the definitions is provided in Multimedia Appendix 1.

### Randomization and Blinding

Participants were randomized to the intervention or the control group in a 1:1 ratio by a researcher who was independent from the recruitment, data collection process, or analyses. The study participants were blinded to the allocation.

### Data Analysis

IBM SPSS Statistics version 20.0 was used for analyzing the data. The quantitative data were analyzed using descriptive statistics such as frequencies, means, and standard deviations (SD). Implementation scores were calculated using the averaging approach, which means that the average scores of the process measures (reach, dose delivered, dose received, and fidelity) were calculated for each function of the intervention. Qualitative data were transcribed verbatim.

## Results

### Reach

**Figure 2:**
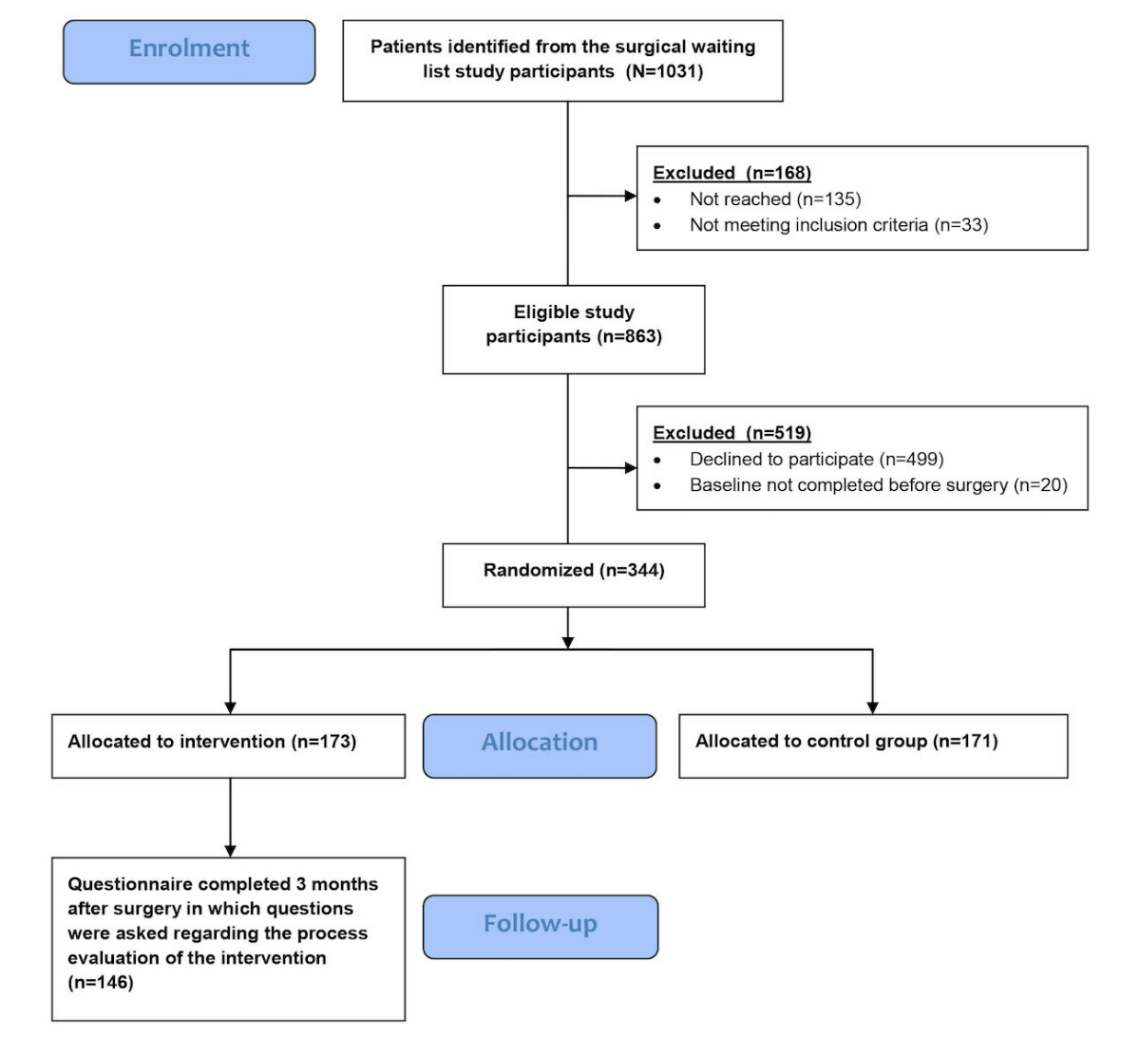
Flow diagram. Only the follow-up results concerning the process evaluation are presented.

During September 2015 and August 2016, 1031 potential participants were identified from the surgical waiting list. The flow of the inclusion process has been described in Figure 2. A total of 344 participants gave consent to participate (39.9%, 344/863); there were no major differences regarding age, gender, and surgical procedure between the participants and nonparticipants.

A total of 173 participants were randomized to the intervention group. The baseline characteristics of these participants are presented in Table 1. In addition, 45.1% were male and the mean age was 51 years; 54 participants underwent adnexal surgery, 68 hernia inguinal surgery (1 open procedure), and 51 a cholecystectomy. The response rate to the questionnaire which was assessed 3 months after surgery, in which questions were asked according to participants’ attitudes regarding the different functions of the intervention and the usage of the app, was 84.4% (146/173). By purposive sampling, participants were selected for an additional interview. After 12 interviews (6 with participants who used the eConsult function and 6 with participants who did not) data saturation was reached.

**Figure 3:**
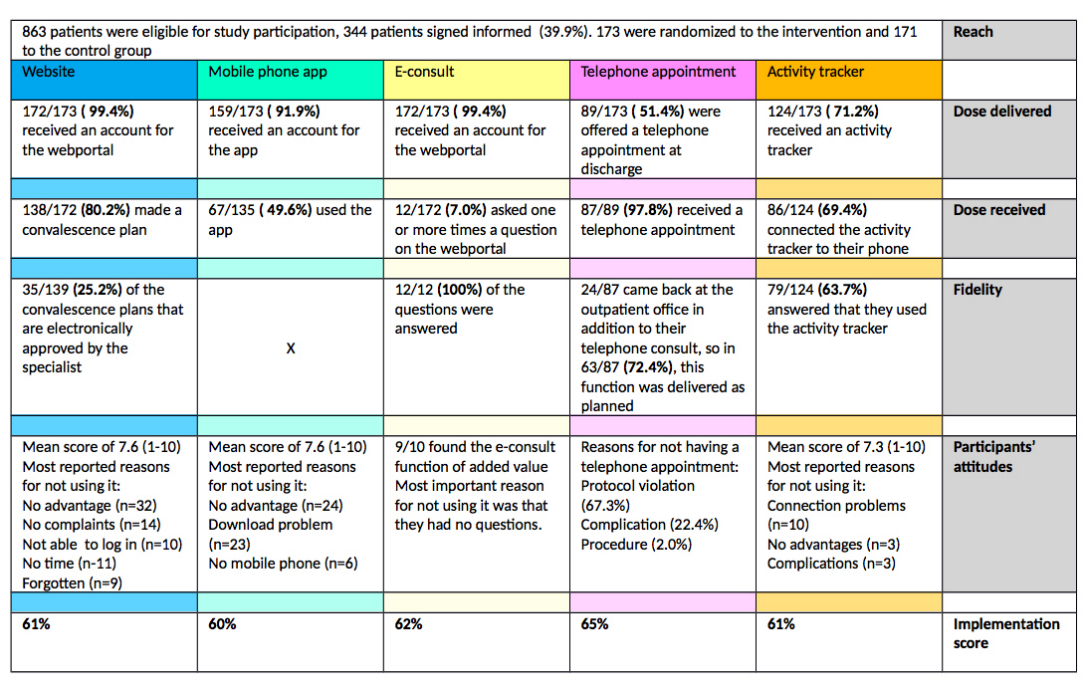
Results of the process evaluation.

The subsample of eConsult users consisted of 2 males and 4 females. Three of them underwent a laparoscopic cholecystectomy, 2 hernia inguinal surgery, and 1 adnexal surgery. The mean age was 39 years. Due to the purposive sampling method, the subsample of participants who did not make use of the eConsult function had the same composition regarding gender and surgical procedure. The mean age in this subsample was 44 years. The other components of the Linnan and Steckler model will be described separately for each part of the intervention and are also presented in Figure 3.

#### Website

##### Dose Delivered

A total of 172 of the 173 participants of the intervention group (99.4%) received an account for the website. One patient did not receive an account due to logistic problems.

##### Dose Received

Of the 172 participants in the intervention group who received an account for the website, 138 (80.2%) developed a convalescence plan on the website.

##### Fidelity

Only 25.2% of the convalescence plans were electronically approved by the medical specialists.

##### Participants’ Attitudes

Participants assessed the website with a mean score of 7.6 on a scale of 1 to 10. Reasons for not (frequently) using the website were that participants reported that they did not see the added value (n=32), had no need for it because they had no complaints (n=14), were not able to log in (n=10), had no time (n=11), had forgotten that there was a website (n=9), underwent another type of surgery (open procedure instead of laparoscopic approach) (n=2), used the app (n=2), did not find the information that they were looking for (n=1), or had no computer (n=1).

**Table 1:** Baseline characteristics.

Variable		Intervention group (N=173)
**Gender, n (%)**		
	Male	78 (45.1)
	Female	95 (54.9)
Age (mean, SD^a^)		51 (12.57)
**Nationality, n (%)**		
	Dutch	171 (98.8)
	Other	2 (1.2)
**Level of education, n (%)**		
	Low	31 (17.9)
	Medium	50 (28.9)
	High	92 (53.2)
**Working situation, n (%)**		
	Employed	132 (76.3)
	Not employed	41 (23.7)
**Type of surgery, n (%)**		
	Laparoscopic adnexal surgery	54 (31.2)
	Laparoscopic hernia inguinal surgery	67 (38.7)
	Open hernia inguinal surgery	1 (0.6)
	Laparoscopic cholecystectomy	51 (29.5)

^a^SD: standard deviation.

#### Mobile Phone App

##### Dose Delivered

Of the 173 participants in the intervention group, 159 (91.9%) had a mobile phone or tablet. All these 159 participants received an account and an information brochure containing instructions on how to download and use the app.

##### Dose Received

Of the 135 participants who received an account for the app and completed the questionnaire 3 months after surgery, 67 (49.6%) answered that they had used the app. A total of 16 participants (23.9%) had used the app only several times, 3 participants (5%) weekly, and 48 participants (72%) several times a week or on a daily basis.

##### Participants’ Attitudes

Participants assessed the app with a mean score of 7.6 on a scale of 1 to 10. Reasons for not using the app were that participants reported that they did not see the added value (n=24), were not able to download the app (n=23), had no mobile phone (n=6), had forgotten it (n=4), suffered from complications (n=4), had no need for it because they had no complaints (n=3), had no time (n=3), or did not find the information they were looking for (n=1). In addition, qualitative data were collected regarding participants’ experiences with using the app. Participants who used the app found it a convenient tool. Several aspects of the app were mentioned as being helpful. One participant stated the following:

The overview of the convalescenceplan in the app was very useful and gave a good picture about what to expect, I resumed my activities quicker because of the app.

Female, 46 years old, laparoscopic adnexal surgery

Another participant stated the following:

The recovery monitor in the app which gives feedback on the speed of my recovery in relation to the convalescenceplan provided me with support and made me feel comfortable.

Female, 38 years old, laparoscopic cholecystectomy

One participant stated the following:

The app was a convenient tool in comparison to the website, because you do not have always your computer quickly available.

Female, 32 years old, laparoscopic cholecystectomy

#### eConsult

##### Dose Delivered

All participants of the intervention group who received an account for the website were automatically provided with the possibility to ask questions to their health care provider by an eConsult (n=172).

##### Dose Received

A total of 12 participants (7.0%) made use of the eConsult function.

##### Fidelity

All 12 questions were answered by the health care providers. Mean time between asking the question and getting a reply was 37 hours.

##### Participants’ Attitudes

The participants who were interviewed mentioned that they had found the eConsult function of added value. The reasons given were that it was an easy or a quick way to ask questions and that they could ask questions while at work. One participant stated the following:

The eConsult function was of added value to me, because I have a busy job so I had no time to call the hospital during office hours. Now I could ask my questions after office hours.

Male, 54 years old, hernia inguinal surgery

However, the participants did not find it useful for all types of questions. One participant stated the following:

The eConsult function on the website is an interesting function, mainly when a quick response could be provided. When you have to wait more than a few days for a response, it will be useless. In addition, for urgent questions (for example high fever) I would have called anyway.

Female, 38 years old, laparoscopic cholecystectomy

Another participant stated the following:

For more complex questions I would have called the hospital because typing emails is not my strongest point.

Male, 60 years old, hernia inguinal surgery

Most of the participants who used the eConsult explicitly mentioned that they would use it again in the future. Most of the eConsult users said that they would not prefer to use the eConsult instead of a (telephone) appointment with the physician, but as an extra facility only. One participant stated the following:

In my opinion the eConsult should not replace the appointment in the outpatient clinic. Personal contact with my doctor is important for me. However, a combination of both would be perfect.

Female, 46 years old, laparoscopic adnexal surgery

Another participant stated the following:

The eConsult should not necessarily replace the appointment in the outpatient clinic. It should be the patient’s choice whether or not he or she prefers to have an appointment.

Female, 40 years old, laparoscopic cholecystectomy

All the participants who had not used the eConsult function mentioned that they had not used it because they had no questions or complaints. Most of them mentioned that they would have used it if they had questions, but one participant mentioned that she would rather have called the hospital in that case:

I have not used the eConsult function because I had no questions or complaints. However, if I had had any questions I rather would have called the hospital because my question would have been quicker answered.

Female, 44 years old, laparoscopic cholecystectomy

#### Telephone Appointment

##### Dose Delivered

A total of 89 participants of the intervention group (51.4%) were offered a telephone appointment at the moment of discharge from the hospital. In 25.5% of the participants, it was unclear whether or not the telephone appointment was provided. The remaining 23.1% (n=40) participants were not offered a telephone appointment.

##### Dose Received

A total of 87 participants (97.8%) received their telephone appointment. The reasons for not receiving the appointment were that that the patient had complaints; therefore, the telephone appointment was replaced by a visit to the outpatient clinic (n=1), and 1 patient requested for an appointment in the outpatient clinic instead of a telephone appointment. The other 61 participants of the intervention group had no postoperative appointment at all (n=37) or had an appointment in the outpatient clinic only (n=49).

##### Fidelity

A total of 24 participants visited the outpatient clinic in addition to their telephone appointment. In 10 participants, this was decided during the telephone appointment, 8 participants visited the outpatient clinic before the telephone appointment because of a complication or complaints, and the reason was unclear in 6 participants.

##### Participants’ Attitudes

There were 49 participants who only came back at the outpatient clinic and thus did not receive a telephone appointment. In 67% of the participants, this was because of a protocol violation, in 22% because of a complication or complaints, in 6% because a procedure had to be performed, and in 2.0% because of a fertility appointment.

#### Activity Tracker

##### Dose Delivered

Of the 173 participants from the intervention group, 124 (71.2%) received an activity tracker. Of the other 55 participants, 14 had no mobile phone and 35 had a mobile phone that was not compatible with the activity tracker.

##### Dose Received

Of the 124 participants who received an activity tracker, 86 (69.4%) connected the activity tracker to their mobile phone.

##### Fidelity

A total of 63.7% of the participants who received an activity tracker have used the activity tracker.

##### Participants’ Attitudes

The activity tracker was assessed with a mean score of 7.3 on a scale of 1 to 10. Reasons for not using the activity tracker were as follows: problems with connecting the activity tracker to their phone (n=10), did not see the added value (n=3), suffered from complications (n=3), had no need for it because they had no complaints (n=1), or because the patient felt too sick to use the activity tracker (n=1). Four out of the 12 participants who were interviewed had not used the activity tracker. Reasons were that their mobile phone was not compatible with the activity tracker or that they felt no need to do it. One participant stated the following:

I felt no need to connect the activity tracker to my phone, that was too much hassle.

Male, 60 years old, laparoscopic hernia inguinal surgery

Most of the participants who used the tracker found it to be a convenient and interesting tool. One participant stated the following:

The activity tracker was a motivator to be more active. It was useful to monitor my movements. However I had to remind myself to wear the activity tracker daily.

Female, 48 years old, laparoscopic adnexal surgery

Another participant stated the following:

The activity tracker was a nice additional tool. It is nice to track how active you are on a day. Sometimes my recovery (displayed on my activity tracker) turned out to be faster than what I was thinking. When I saw for example my activity status of two weeks earlier compared to my current status, I realized that my recovery was going faster than I expected. It was a motivational tool for me.

Female, 40 years old, laparoscopic cholecystectomy

One participant stated the following:

The activity tracker was easy in use and it was very nice to track my activities. It worked motivating for me. I have moved more to reach my goal. [Female, 32 years old, laparoscopic cholecystectomy].

#### Implementation Scores

The implementation scores of the different functions of the intervention are presented in Figure 3. They coincide very closely, ranging between 60% and 65%.

## Discussion

### Principal Findings

In this process evaluation, the implementation process of a perioperative eHealth intervention, comprising a website, app, activity tracker, eConsult function, and a telephone appointment 2 weeks after surgery, was evaluated. The implementation scores of the different functions of the intervention were fair and ranged between 60% and 65%. The website, app, and activity tracker were assessed with a mean score of 7.3 to 7.6 on a scale of 1 to 10. Twelve study participants were interviewed about the eConsult function; almost all rated it as being of additional value when combined with the usual care.

### Interpretation of the Results

The implementation scores were fair, which was caused by the fact that some of the functions of the intervention scored surprisingly low regarding the components of the Linann and Steckler model. In our opinion, there may be three possible reasons for this. First, there was a lack of continuity in providing the intervention to the participants. Because the 173 participants from the intervention group were included in 7 different centers, a mean of 25 participants were included per center in a 1 year period. It is likely that this low volume of patients would have caused the fair implementation and that if the intervention would be implemented in clinical practice outside study setting and, as a consequence, the intervention would be provided to every patient routinely, scores will be much higher. In our opinion, this lack of routine will be the major explanation for the fact that only 25.2% of the convalescence plans were electronically approved by the specialist and only 51.4% were offered a telephone appointment. Another possible explanation for the fair implementation scores is that there is really no need for this specific function. We think that this could be the case in the eConsult function, which was only used in 7% of the participants to whom it had been offered. In the additional interviews we performed, almost all participants answered that they would have used the function in the case they had questions; however, they had no questions. A final explanation could be that participants were hampered by technical barriers to use the intervention. Especially for the activity tracker, the interviews showed that for some participants the different steps that had to be undertaken to install the tracker were a barrier for using it. This could be overcome by a helpdesk providing assistance in this. However, the procedure itself can also be simplified and more easy to use. On the basis of this finding, we can improve the procedures related to installing the activity tracker.

### Comparison With Prior Work

In 2014, Bouwsma et al published a process evaluation about the eHealth intervention, which was the base for the development of the eHealth intervention evaluated in this study [15]. Bouwsma reported an implementation score of the eHealth intervention of 80.3%, which was between 15% and 20% higher than the implementation scores of our study. There are some possible explanations for the difference in scores. First of all, and in our opinion the most important one, is the degree of involvement of the researcher in both studies regarding motivating the study participants to use the intervention. For example, the eHealth intervention which was evaluated in the process evaluation of Bouwsma et al was provided to the study population, and when the research team signaled that is was not being used by the study participant, the researcher contacted the study participant to offer assistance. In addition, when the convalescence plan was not approved by the medical specialist, the specialist was contacted by the researcher to bring it to his or her attention. In this study, we tried to limit the involvement of the researcher to a minimum to have a realistic perspective on the actual implementation, including the potential barriers. After the intervention was delivered to the participant, the research team only provided assistance when the study participant contacted them. It was decided to do so as we wanted to create a situation that was most comparable with the situation in which the intervention would be implemented in the future with a helpdesk (outside study setting). In our opinion, this is of great importance as the implementation of eHealth interventions has proven to be a difficult process; so, when we aim to evaluate the barriers of implementation, we should evaluate the intervention in a situation that is as similar to the future situation as possible.

### Strengths and Limitations

One of the strengths of the study was the extensiveness in which the process evaluation was performed. This is because the individual functions of the intervention were evaluated separately. By evaluating the individual functions of the intervention, important information was generated according as to what makes the intervention more or less effective, which can be of assistance in the future for the purpose of adapting the intervention. Another strength of the study is the high response rate (84.4%) to the questionnaire that was used to assess participants’ attitudes regarding the intervention 3 months after surgery. In addition, the data collection process consisted of several components. Quantitative data containing objective data from a weblog and a logistic database as well as more subjective data assessed by questionnaires were collected, and in addition, qualitative data were collected by interviews with a sample of the study population. However, qualitative data were only collected in a small subsample (n=12) of the study population, and therefore, these data were only presented as an example descriptively and should be interpreted with caution. This study also has some limitations. The most important one is the fact that we have not collected information regarding reasons for nonparticipation. As 60% declined to participate, it would be very valuable to know whether this was because of the study setting and associated burden or because of the fact that the patients had no need for a perioperative eHealth intervention. In our opinion, it is not likely that the latter reason was the major reason for nonparticipation. We performed a survey study 1 year earlier in one of the hospitals that also participated in this study. In this study, patients who had undergone adnexal, hernia inguinal, or a cholecystectomy were also included, and 78% of them indicated that they had felt the need for an eHealth program during their perioperative course [16]. Second, although we think that it is a strength that we evaluated the different functions of the intervention separately, it was difficult to define some components of the Linann and Steckler model regarding the functions of the intervention, for example, the definition of “dose received” of the eConsult function. The nominator was defined as *Participants who asked a question on the Web portal* and the denominator as *Participants who received an account for the Web portal.* Ideally, the denominator would be only the participants who had a question; however, we did not measure this. A final limitation of the study is the questionable manner in which the implementation scores are calculated. This is well illustrated by the fact that we have calculated nearly five identical implementation scores, whereas the individual components of each function of the intervention differed considerably. For example, dose received was 7.0% for the eConsult function and 97.8% for the telephone appointment; however, the implementation scores were nearly the same (62% and 65%, respectively). We used the averaging approach; however, Baranowski et al recommend that the implementation score has to be the result of the product of reach, dose, and fidelity [17]. As the calculation of the implementation score is doubtful, we should be careful with interpreting these scores.

### Clinical Implications and Future Research

This study has several important implications. First, the results are of great relevance when interpreting the results regarding the effectiveness evaluation of this study, which will become available in the future. Second, it may be helpful for future research regarding the implementation of these types of eHealth interventions. Unless some eHealth interventions are proven to be effective, the usage in daily practice of the intervention fails most of the time. Therefore, research evaluating the barriers and facilitators for implementing eHealth interventions should be carried out. In this study, we evaluated the feasibility of the intervention. Another study is needed to evaluate implementation barriers in daily practice on a wide scale, because in this study, the intervention was applied in a study setting. This may have influenced the results, mainly the results regarding the dose-delivered component, because the intervention was delivered by the researcher who was likely more involved in the process than, for example, a health care provider who had to deliver the intervention in the future outside the research setting. Finally, the study setting may also have influenced the results, as only 39.9% of the assessed participants gave consent to participate and likely the participants who participated were not a good reflection of the overall population. More qualitative research should therefore be performed in the future focusing on all stakeholders, such as patients, caregivers, and policymakers, that are not involved in an effectiveness study.

### Conclusions

In conclusion, participants were overall satisfied with the intervention. However, the implementation scores of the different functions of the intervention were fair. More research is needed to evaluate the barriers and facilitators for implementation of this perioperative eHealth intervention before it can be implemented outside the study setting.

## Appendix

Multimedia Appendix 1 Description of the outcome measures.

Multimedia Appendix 2 CONSORT-EHEALTH checklist (V 1.6.1).

## Abbreviations

CONSORT: Consolidated Standards of Reporting Trials
eConsult: electronic consultation
eHealth: electronic health
SD: standard deviation
