# Supplementary material for: A Perioperative eHealth Program to Enhance Postoperative Recovery After Abdominal Surgery: Process Evaluation of a Randomized Controlled Trial
Source: J Med Internet Res. 2018 Jan 2;20(1):e1. doi: 10.2196/jmir.8338 (PMC5770580; doi:10.2196/jmir.8338)
Supplement: Multimedia Appendix 1 [file jmir_v20i1e1_app1.pdf]

Reach

Reach was defined as the proportion of the target population who were assessed for eligibility, met the inclusion criteria, signed informed consent and were randomized to the intervention or control group. A logistic database was completed by the researcher to assess these measurements.

Website

Dose Delivered

When participants were allocated to the intervention group they received an account for the website by e-mail provided by the researcher. Dose delivered was calculated by dividing the number of participants who received an account for the website by all participants of the intervention group. This was measured with a logistic database, in which the researcher registered when an account was sent to the participant.

Dose Received

One of the most important functions of the website was the possibility to make a personalized convalescence plan. This convalesce plan was based on multidisciplinary convalescence recommendations which were developed through a modified Delphi study [14, 15]. Participants could select the activities which were important for them in daily life and subsequently a convalescence plan was generated automatically by the computer using algorithms. Because this was the most important tool of the website, dose received was calculated by dividing the number of participants who made a convalesce plan by the number of participants of the intervention group who received an account for the website. This was assessed by a weblog. The researcher had access to this weblog in which the most important user statistics of the website were visible.

Fidelity

When a patient of the intervention group generated a convalescence plan, his or her surgeon received an e-mail the day after surgery in which was asked if a complication occurred during the surgical procedure. Reason for this was that the convalescence plan was only developed for participants who underwent a surgical procedure without peri-operative complications . When no complications occurred the surgeon was requested to approve the convalescence plan. When the surgeon indicated that a complication had occurred, the patient was automatically informed that the convalescence plan was no longer applicable to him or her. Fidelity was therefore calculated by dividing the number of patients with convalescence plans that were electronically approved by the medical specialists by the all patients who made a convalescence plan. This was measured by the weblog.

Participants' Attitudes

Participants' attitudes regarding the website were assessed in a questionnaire three months after surgery. Participants were asked to assess the website on a scale from 1 to 10. In addition, reasons for not using the website were assessed.

Mobile Phone Application

Dose Delivered

The mobile phone application contained the same information and functions as the website. When participants gave consent to participate in the study, they were asked if they had a smartphone or tablet. This was asked because only the participants with a smartphone or tablet had to receive the instruction about how to download, install and use the mobile phone application (app). Dose delivered of the app was calculated by dividing the number of participants from the intervention

group who had a smartphone or tablet and thus received an account for the app including the instructions by all participants of the intervention group. This was assessed with the logistic database.

Dose Received

Dose received was the result of dividing the participants who used the app by the participants who received an account for the app. It was not possible to measure this by user statistics, therefore this was asked in the questionnaire three months after surgery.

Fidelity

Fidelity of the app was not defined because there was no prescribed description about how the app had to be used.

Participants' Attitudes

In the same questionnaire as described above, participants who used the app were asked to assess the app on a scale from 1 to 10. Participants who answered that they did not use the app were asked about the reasons for not doing so. In addition a sample of study participants were interviewed and asked about their opinion about the app.

EConsult

Dose Delivered

After surgery, a button appeared on the website with the description 'I have a question'. By clicking on this button participants could fill in their question and subsequently the question was sent to a health care professional of the hospital in which the patient underwent surgery. Because the eConsult function was provided on the website, the definition of dose delivered regarding the eConsult was the same as for the website.

Dose Received

Dose received was calculated by dividing the number of participants who asked a question by way of an eConsult one or more times by the number of participants of the intervention group who received an account for the website. This was measured by the user statistics displayed in the weblog.

Fidelity

The proportion of the questions asked which were answered by the health care professional was defined as fidelity. This was also measured by the user statistics of the weblog. In addition it was possible to calculate the mean timeframe in which the questions were answered.

Participants' Attitudes

Participants' attitudes regarding the eConsult were assessed by interviews with a selection of the study participants; a selection of participants who used the eConsult function and a selection who did not. Questions were asked about their opinion regarding the eConsult function, reasons for using or not the eConsult function and regarding future applicability of the eConsult.

Telephone Appointment

Dose Delivered

After discharge, a telephone appointment was offered to the participants of the intervention group instead of an appointment in the outpatient clinic. Reason for this was that these participants also had the possibility to use the eConsult function. Dose delivered was defined as the proportion of the participants of the intervention group who were offered a telephone appointment at discharge.

Dose Received

Dose received was calculated by dividing the number of participants who were called by the

participants who were offered a telephone appointment at the time of discharge. Reasons for not having the appointment were evaluated by reviewing the medical file.

Fidelity

Fidelity was calculated by dividing the number of participants who came back to the outpatient clinic in addition to their telephone appointment by the participants who had a telephone appointment. Reasons for this were assessed by checking the medical file.

Participants' Attitudes

Reasons for not having a telephone appointment were defined as participants' attitudes and were assessed by a questionnaire three months after surgery.

Activity Tracker

Dose Delivered

Only the group of participants from the intervention group who had a smartphone or tablet which was compatible with the activity tracker received an activity tracker. This proportion of the participants was defined as dose delivered.

Dose Received

Dose received was calculated by dividing the number of participants who connected the activity tracker to their smartphone by the participants who received an activity tracker.

Fidelity

Participants were asked to wear the activity tracker during the week before surgery, during the first three weeks after surgery and in the sixth week after surgery. Fidelity was defined as the proportion of the participants who received the activity tracker, who used the activity tracker as intended.

Participants' Attitudes

Participants were asked to assess the usability of the activity tracker during the questionnaire, three months after surgery. They were asked to access the tracker with a number on a scale from 1 to 10 and to give reasons for not wearing the activity tracker. In addition the sample of participants who were interviewed were also asked about their opinion regarding the activity tracker and about their reasons for not using the tracker.
